# Mitochondrial Activation and Reactive Oxygen-Species Overproduction during Sperm Capacitation are Independent of Glucose Stimuli

**DOI:** 10.3390/antiox9080750

**Published:** 2020-08-14

**Authors:** David F. Carrageta, Bárbara Guerra-Carvalho, Mário Sousa, Alberto Barros, Pedro F. Oliveira, Mariana P. Monteiro, Marco G. Alves

**Affiliations:** 1 Department of Microscopy, Laboratory of Cell Biology, Unit for Multidisciplinary Research in Biomedicine (UMIB), Institute of Biomedical Sciences Abel Salazar (ICBAS), University of Porto, 4050-313 Porto, Portugal; davidcarrageta@gmail.com (D.F.C.); barbaraggcarvalho@gmail.com (B.G.-C.); msousa@icbas.up.pt (M.S.); 2 Centre for Reproductive Genetics Alberto Barros, 4100-012 Porto, Portugal; abarros@med.up.pt; 3 Department of Genetics, Faculty of Medicine, University of Porto, 4200-319 Porto, Portugal; 4 i3S—Instituto de Investigação e Inovação em Saúde, Universidade do Porto, 4200-135 Porto, Portugal; 5 QOPNA & LAQV, Department of Chemistry, University of Aveiro, 3810-193 Aveiro, Portugal; pfobox@gmail.com; 6 Clinical and Experimental Endocrinology, Unit for Multidisciplinary Research in Biomedicine (UMIB), Institute of Biomedical Sciences Abel Salazar (ICBAS), University of Porto, 4050-313 Porto, Portugal; mpmonteiro@icbas.up.pt

**Keywords:** capacitation, glucose, mitochondrial activity, oxidative stress, ROS, spermatozoa

## Abstract

Spermatozoa capacitation is a complex process that requires specific ionic and energetic conditions to support biochemical alterations leading to motility hyperactivation. However, human sperm capacitation is still poorly understood. Herein, we studied the effects of glucose on human sperm capacitation. Healthy men seminal samples (*n* = 55) were submitted to a density gradient centrifugation and incubated in capacitating conditions in the absence or presence of increasing glucose concentrations (0, 5.5, 11, and 22 mM). Viability and total motility were accessed. Phosphotyrosine levels were measured. Mitochondrial activity and endogenous ROS production were evaluated. Oxidative stress-induced damage was analyzed. Culture media was collected and analyzed by ^1^H-NMR. Our results show that glucose is essential for human sperm capacitation and motility. Notably, we observed that mitochondrial activity increased even in the absence of glucose. This increased mitochondrial activity was followed by a ROS overproduction, although no oxidative stress-induced damage was detected. Our results show that glucose is essential for capacitation but mitochondrial activation is independent from its stimuli. ROS overproduction may take part on a finely regulated signaling pathway that modulates or even activates capacitation. Taken together, our results constitute a paradigm shift on human sperm capacitation physiology.

## 1. Introduction

Capacitation is a complex and finely regulated process of structural and functional alterations that occur in mammalian spermatozoa essential for oocyte fertilization. Sperm capacitation occurs naturally in the female reproductive tract and involves membrane modifications, several proteins phosphorylation and activation of signaling cascades. During capacitation, the sperm acquires the ability to undergo acrosome reaction and achieves a state of hyperactivation, characterized by an increased motility with asymmetric beat and increased flagellar bend amplitude that improves sperm swimming ability and aids zona pellucida of the oocyte penetration [1]. A successful capacitation requires a specific ionic environment, a fine regulation of signaling cascades, and energy management in order to support the biochemical alterations leading to the hyperactivation state [2,3]. The conditions for sperm capacitation were firstly described in 1951 and its discovery allowed the development of in vitro fertilization (IVF) [4,5,6]. In the female reproductive tract, human spermatozoa are exposed to a high concentration of HCO_3_^−^, albumin, Ca^2+^, and an alkaline pH, which are essential for phosphorylation cascade activation and hyperactivated motility [7]. On an energetic perspective, glucose is considered the preferential energy substrate for human sperm capacitation and hyperactivation [8]. Sperm hyperactivation and subsequent increased motility have high ATP demands, whereas intracellular ionic balance maintenance also requires active transport and, consequently, increased ATP production for its sustainability [3,9].

In human spermatozoa, glycolysis and mitochondrial oxidative phosphorylation are the two major metabolic pathways that are activated for ATP generation from glucose [10]. Human sperm are very specialized and unique cells, exhibiting a unique metabolism regulated by specific enzyme isoforms [11]. For instance, glycolysis and mitochondrial respiration occur in distinct subcellular locations. Mitochondria are localized exclusively in the sperm midpiece, whereas glycolytic enzymes predominate in the flagellum [2]. However, there is no consensus concerning which one is the main ATP source in human spermatozoa. While some authors proposed glycolysis to be the exclusive ATP source [12], others defended aerobic ATP production by sperm mitochondria to be essential for supporting human sperm capacitation and hyperactivated motility [13,14,15]. In support of the latter, some studies reported an increase in mitochondrial respiration during capacitation, which was positively associated with hyperactivated motility [16,17]. Nevertheless, mitochondria are also a source of reactive oxygen species (ROS), which at low concentrations play physiological roles [18]. On the other hand, high ROS concentration leads to protein and lipid damage [19,20]. Taken together, the physiology and bioenergetics of human sperm capacitation are still poorly understood. The interaction between glucose stimuli as energy source and mitochondria functioning, as well as the role of ROS remain to be clarified.

Herein, our aim was to study the effects of glucose on human sperm capacitation. For this purpose, human spermatozoa were incubated in vitro under capacitation conditions with increasing glucose concentrations (5.5, 11 and 22 mM) or without this energetic substrate. The effects of increasing glucose concentrations on human sperm viability and motility, protein phosphorylation levels and metabolism, were then characterized. As capacitation is associated with increased mitochondrial activity, we have also analyzed mitochondria membrane potential, ROS production and biomarkers for oxidative stress-induced damage.

## 2. Materials and Methods

### 2.1. Chemicals

All chemicals were purchased from Sigma-Aldrich (St. Louis, MO, USA) unless stated otherwise.

### 2.2. Ethical Approval

Human seminal samples were obtained from men seeking infertility treatment at the Centre for Reproductive Genetics A. Barros (Porto, Portugal). Patients selection, clinical study, and diagnostic semen analysis were performed at the clinic. All procedures were conducted in accordance with the ethical guidelines for human samples research and informed and written consent was obtained from all individual donors included in the study. This study did not involve experiments on humans or animals, as only donated samples of surplus fresh ejaculated semen samples collected for diagnostic purposes were used. The approval of the Ethics Committee and the Declaration of Helsinki, revised in Tokyo 2004, on human experimentation does not apply to this work. The Centre for Reproductive Genetics A. Barros infertility clinic’s procedure are under the provisions of the National Medically Assisted Procreation Act (Law of 2017) and overseen by the National Council for Medically Assisted Procreation (CNPMA-2018). According to these rules and guidelines, the use of clinical databases and patient biological material for diagnosis and research may be used without further ethical approval, under strict individual anonymity, and after patient written informed consent. Regarding the use of semen samples for laboratory experimentation at ICBAS-UP, the Ethics Committee authorization number is Project: 2019/CE/P017 (266/CETI/ICBAS).

### 2.3. Human Sperm Samples

Normozoospermic subjects were recruited among couples seeking infertility treatment at the medically assisted procreation clinic. Samples from healthy adult male donors (mean age of 36 ± 5 years old) were obtained by masturbation into specific sterile containers 3 days after sexual abstinence for a diagnostic semen analysis. Fresh ejaculated semen samples were left at 37 °C until complete liquefaction, then analyzed according to the guidelines of the World Health Organization (WHO) [21]. Only samples of normozoospermic subjects (*n* = 55) were selected for this study. A standard discontinuous density gradient centrifugation was performed on the selected 55 samples using the Gems^®^ Sperm Wash Gradient Set (45% and 90%) (Merck KGaA, Darmstadt, Germany) according to the manufacturer’s instructions. Solutions were stabilized at room temperature before the gradient was constituted. All procedures were performed at room temperature. Only a maximum volume of 1.5 mL of seminal sample was placed on each gradient. Samples with higher volumes were separated in different gradients. The resultant pellet, containing the spermatozoa fraction with higher percentage of motility and viability [22,23], was collected and washed 2 times in sterile PBS followed by a centrifugation of 500× *g* for 5 min.

### 2.4. Experimental Groups

For the purpose of studying the effects of increasing glucose concentrations on human sperm capacitation, retrieved spermatozoa from each sample isolated through density gradient centrifugation were equally separated into four groups. As a low concentration of spermatozoa is retrieved, not all samples were used for all experiments. Therefore, samples were randomly distributed among the experiments. A maximum of 15 million spermatozoa were incubated in a 5% CO_2_ atmosphere at 37 °C in 1 mL of a modified Biggers, Whitter and Whittingham (BWW) medium for the stimulation of sperm capacitation (in mM: 94.5 NaCl, 4.8 KCl, 1.7 CaCl_2_, 1.17 KH_2_PO_4_, 1.22 MgSO_4_, 20 HEPES, 24 NaHCO_3_, 0.4 BSA, pH = 7.4) supplemented with increasing glucose concentrations (5.5, 11 and 22 mM). To study the effects of glucose absence, a group was incubated in BWW medium only. After a 6 h incubation, samples were centrifuged and media was collected for ^1^H-NMR analysis. Spermatozoa were then washed 2 times in PBS and a fraction of treated spermatozoa was collected either for viability, total motility, mitochondrial membrane potential, or endogenous ROS content analysis. The remaining spermatozoa were centrifuged and the pellet collected for protein analysis. A spermatozoa fraction obtained following the gradient density centrifugation was collected and used as reference group (T = 0 h). Results concerning the reference group were obtained 90 min after obtaining the samples.

### 2.5. Sperm Viability and Total Motility Analysis

Sperm membrane integrity was analyzed using the eosin/nigrosin staining protocol as viability indicator (*n* = 15 for each condition), as previously described [24]. A total of 5 µL of spermatozoa suspensions obtained before and after treatments were mixed with 5 µL of 0.5% eosin-nigrosin stain and smeared on a glass microscope slide. The percentage of viable and nonviable spermatozoa was determined by counting a total of 200 spermatozoa per slide in continuous random fields under a light microscope (1000× magnification). White spermatozoa were considered viable whereas pink-stained spermatozoa, indicating loss of membrane integrity, were considered nonviable.

Sperm total motility (*n* = 20 for each condition) was assessed using a Makler counting chamber (Sefi Medical Instruments, Haifa, Israel), according to the manufacturer’s recommendations. A drop of spermatozoa suspensions (10 µL) was placed in the center of the chamber and carefully covered to ensure an evenly sperm distribution. The experiment was performed by the same user and the same criteria was applied between samples. Sperm motility was then assessed in 20 squares using an optical microscope (200× magnification). New drops were placed on the chamber and the process was repeated until a total of 100 sperm cells per sample were counted. Results are expressed in percentage of motile spermatozoa.

### 2.6. Proton Nuclear Magnetic Resonance (^1^H-NMR)

^1^H-NMR spectra were acquired using a 600 MHz Varian spectrometer (Varian, Inc., Palo Alto, CA, USA) equipped with a 3-mm indirect detection probe, as previously described [25]. A total of 180 µL of medium of each condition (*n* = 15) was analyzed. Samples were diluted with a sodium fumarate and deuterated water solution (final concentration of 2 mM in 225 µL), which was used as internal reference (6.50 ppm) to quantify the following metabolites present in the medium (multiplet, ppm): lactate (doublet, 1.33), acetate (singlet, 1.90), and malate (double doublet, 2.37). Spectra were manually phased and baseline corrected. Chosen metabolites peaks were integrated using the NUTS-Pro NMR software (Acorn NMR, Inc, Fremont, CA, USA). Results are expressed as nmol/10^6^ spermatozoa.

### 2.7. Sperm Capacitation Analysis

Human spermatozoa lysates were prepared from the resulting pellets. Pellets were resuspended in RIPA buffer and incubated for 15 min at 4 °C. The samples were then centrifuged (14,000× *g* for 20 min) and total protein concentration was quantified using a BCA Protein Assay Kit (Thermo Scientific, Waltham, MA, USA) according to the manufacturer’s instructions.

The phosphorylation state of spermatozoa proteins was used as a biomarker to evaluate sperm capacitation [26]. Protein samples were diluted to a final concentration of 50 ng/µL and transferred to a PVDF membrane using a slot blot technique in a Hybri-slot manifold system (Biometra, Göttingen, Germany). A Ponceau S staining solution (MB19201, NZYTech, Lisboa, Portugal) was used for total protein normalization. The resulting membranes (*n* = 6 for each condition) were incubated overnight at 4 °C with a mouse anti-phosphotyrosine monoclonal antibody (1:1000, Clone 4G10, #05-321, Merck Millipore, Temecula, CA, USA). Membranes were then incubated with a goat anti-mouse IgG antibody (1:5000, AP308P, Sigma-Aldrich, St. Louis, MO, USA). Blots were visualized with Clarity™ Western ECL Substrate (Bio-Rad, Hercules, CA, USA) and read using a Bio-Rad ChemiDoc XR system (Bio-Rad, Hercules, CA, USA). Densities from each band were quantified using Image Lab Software (Bio-Rad, Hercules, CA, USA).

### 2.8. JC-1 Assay for Mitochondrial Membrane Potential

The mitochondrial membrane potential of spermatozoa was evaluated using the fluorogenic dye JC-1 (T3168, Invitrogen™, Carlsbad, CA, USA). In brief, 10^6^ spermatozoa were collected before and after treatments and incubated in a JC-1 solution (1 µg/mL in PBS) at 37 °C for 30 min. Spermatozoa incubated in a 20% DMSO solution were used as a positive control. Then, cells were centrifuged at 500× *g* for 5 min and washed 2 times with PBS. Sperm cells were then transferred into a 96-well plate and monomers (ex 485/530 nm; excitation/emission) and J-aggregates (535/590 nm; excitation/emission) fluorescence read on a Synergy™ H1 multi-mode microplate reader (BioTek, Winooski, VT, USA). The ratio between JC-1 J-aggregates/monomers was calculated (*n* = 15 for each condition) and used as an indicator of mitochondrial membrane polarization, where an increased/decreased ratio was considered indicative of hyperpolarization/depolarization, respectively [27]. Results are expressed in fold variation to the reference group (T = 0 h).

JC-1 J-aggregates/monomers fluorescence were also observed in a Nikon Eclipse E400 microscope equipped with a DS-Fi3 microscope camera, a Y-FL Epi-Fluorescence Attachment, and HB-10103AF Super High Pressure Mercury Lamp Power Supply (Nikon, Shinagawa, Tokyo, Japan). Nuclei were stained with Hoechst 33,342 (H3570, Thermo Scientific, Waltham, MA, USA). Pictures were captured with a 1000× magnification and a DAPI filter (395/425 nm; excitation/emission) was used for observing the nuclei, FITC filter (480/510 nm; excitation/emission) for JC-1 monomer, and G-2A filter (510/575 nm; excitation/emission) for JC-1 J-aggregates. All pictures were captured with the same exposition time and gain. The obtained pictures were processed using the NIS-Elements Br 4.60.00 software (Nikon, Shinagawa, Tokyo, Japan).

### 2.9. Detection of Intracellular Reactive Oxygen Species (ROS)

Total ROS produced by spermatozoa were evaluated with the fluorogenic dye CM-H_2_DCFDA (C6827, Invitrogen™, Carlsbad, CA, USA), according to the manufacturer’s instructions. CM-H_2_DCFDA is a chloromethyl derivate of H2DCFDA, whose oxidation originates a fluorescent adduct that can be used as an indicator for intracellular ROS in cells. Briefly, 10^5^ spermatozoa were collected before and after glucose treatments and incubated in a CM-H_2_DCFDA solution (5 µM in PBS) at 37 °C for 30 min. Spermatozoa incubated in a 0.1% H_2_O_2_ solution was used as a positive control. Sperm cells were then transferred into a 96-well plate and fluorescence (495/529 nm; excitation/emission) read on a Synergy™ H1 multi-mode microplate reader (BioTek, Winooski, VT, USA). Results are expressed in fold variation to the reference group (T = 0 h).

### 2.10. Evaluation of Oxidative Stress-Related Damage

The potential oxidative stress-related damage was evaluated on the spermatozoa through the analysis of protein oxidation (*n* = 15 for each condition), lipid peroxidation (*n* = 15 for each condition), and protein nitration (*n* = 8 for each condition) by the slot blot technique and using specific antibodies, according to methods previously described [28]. Briefly, for the analysis of protein oxidation the content of carbonyl groups was assessed. Protein samples of 5 µg were derivatized using 2,4-dinitrophenylhydrazine (DNPH) to obtain 2,4-dinitrophenyl (DNP) and then diluted to a final concentration of 1 ng/µL. For lipid peroxidation and protein nitration analysis, protein samples were diluted to a concentration of 50 ng/µL. The slot blot technique was performed using a Hybri-slot manifold system (Biometra, Göttingen, Germany). A Ponceau S staining solution (MB19201, NZYTech, Lisboa, Portugal) was used for total protein normalization. The resulting PVDF membranes were incubated overnight at 4 °C with a rabbit anti-DNP antibody (1:5000, D9656, Sigma-Aldrich, St. Louis, MO, USA), goat anti-4-hydroxynonenal antibody (1:5000, AB5605, Merck Millipore, Temecula, CA, USA), and rabbit anti-nitro-tyrosine antibody (1:1000, 9691S, Cell Signaling Technology, The Netherlands), respectively. Membranes were then incubated with a rabbit anti-goat IgG antibody (1:5000, AP107P, Sigma-Aldrich, St. Louis, MO, USA) or goat anti-rabbit IgG antibody (1:5000, AP307P, Sigma-Aldrich, St. Louis, MO, USA). Blots were visualized with Clarity™ Western ECL Substrate (Bio-Rad, Hercules, CA, USA) or Vistra ECF™ Substrate (RPN5785, GE Healthcare, Chicago, IL, USA) and read using a Bio-Rad ChemiDoc XR system (Bio-Rad, Hercules, CA, USA). Densities from each band were quantified using Image Lab Software (Bio-Rad, Hercules, CA, USA). Results are expressed in fold variation to the reference group (T = 0 h).

### 2.11. Statistical Analysis

Experimental results are presented as mean ± standard error of mean (SEM). Statistical analysis was performed in GraphPad Prism 6 (GraphPad Software, San Diego, CA, USA). Data were tested for normal distribution with a Kolmogorov-Smirnov test. Statistical differences were determined by a repeated measures ANOVA or one-way ANOVA. *p* < 0.05 was considered significantly different.

## 3. Results

### 3.1. Glucose is Essential for Human Sperm Viability Maintenance and Capacitation

**Figure 1:**
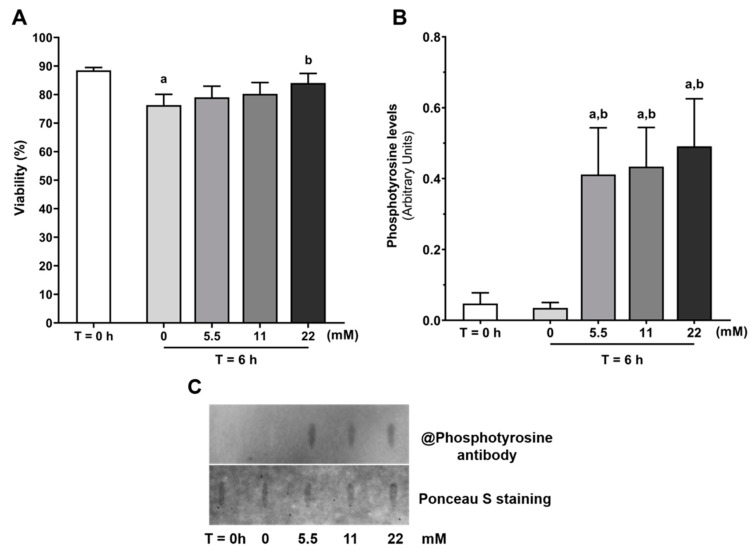
Evaluation of human spermatozoa viability as determined by eosin-nigrosin staining (**A**) and mean tyrosine residues phosphorylation levels (**B**) at baseline (T = 0 h) and without or with glucose (0, 5.5, 11 and 22 mM) stimuli for 6 h in capacitation conditions. (**C**) Representative slot blot membrane. Results are expressed as mean ± SEM ((**A**) *n* = 15 for each condition, (**B**) *n* = 6 for each condition). Significantly different results are expressed as: (**a**) Relative to T = 0 h group; (**b**) Relative to 0 mM glucose group (T = 6 h).

Following the 6 h incubation with increasing glucose concentrations (5.5, 11 and 22 mM) or absence of exogenous energy substrates in capacitating conditions, the percentage of viable spermatozoa were assessed through an eosin-nigrosin staining (Figure 1A). We observed that in the absence of glucose (as well as any other exogenous energy substrate), the percentage of viable spermatozoa decreased (76 ± 4%) as compared with the T = 0 h group (89 ± 1%, *p* = 0.003). No differences were observed between glucose exposed groups when compared to T = 0 h or between glucose concentrations (79 ± 4%; 80 ± 4%; 84 ± 3% after treatment with 5.5, 11 and 22 mM of glucose, respectively). Interestingly, spermatozoa treated with the highest glucose concentration (22 mM) presented a higher viability when compared with the group treated in the absence of glucose (*p* = 0.046).

Tyrosine residues phosphorylation is a common biomarker to evaluate human sperm capacitation. Tyrosine residues phosphorylation levels were determined through slot blot technique (Figure 1B,C). Glucose treatment increased human spermatozoa tyrosine residues phosphorylation at all concentrations (0.412 ± 0.132 arbitrary units; 0.434 ± 0.111 arbitrary units; 0.491 ± 0.134 arbitrary units, after treatment with 5.5, 11 and 22 mM of glucose, respectively) when compared to the T = 0 h group (0.048 ± 0.030 arbitrary units; *p* = 0.021, *p* = 0.016, *p* = 0.005, respectively) and to the no glucose group (0.035 ± 0.015 arbitrary units; *p* = 0.017, *p* = 0.012, *p* = 0.004, respectively). No differences were observed between the glucose groups or between the group with no glucose and the T = 0 h group.

### 3.2. Glucose Exposure Increases Human Sperm Total Motility

**Figure 2:**
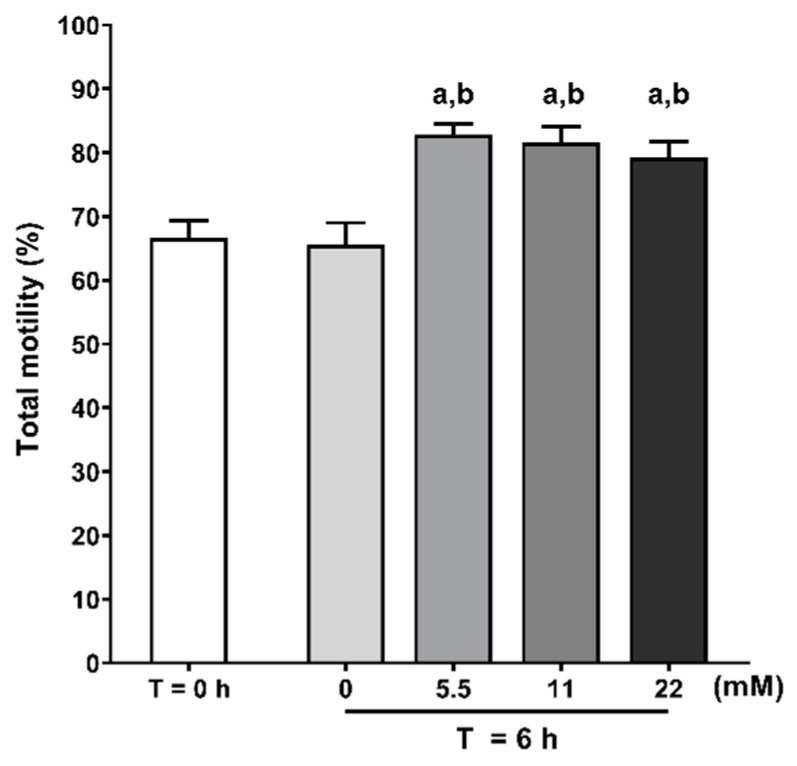
Evaluation of human spermatozoa total motility at baseline (T = 0 h) and without or with glucose (0, 5.5, 11 and 22 mM) stimuli following 6 h incubation in capacitation conditions. Results are expressed as mean ± SEM (*n* = 20 for each condition). Significantly different results are expressed as: (**a**) Relative to T = 0 h group; (**b**) Relative to 0 mM glucose group (T = 6 h).

Human sperm capacitation is characterized by the increased conversion of energy substrates into mechanical energy, resulting in increased motility. In order to study the effects of increasing glucose concentrations (5.5, 11, and 22 mM) or absence of exogenous energy substrates on human spermatozoa motility during capacitation, we evaluated the percentage of motile spermatozoa using a Makler chamber (Figure 2). We observed that the exposure to glucose increased the percentage of motile spermatozoa at all tested concentrations (83 ± 2%; 82 ± 3%; 79 ± 3%, after treatment with 5.5, 11 and 22 mM of glucose, respectively) when compared to the T = 0 h group (67 ± 3%; *p* < 0.001, *p* < 0.001, *p* = 0.024, respectively) and to the no glucose group (66 ± 4%; *p* = 0.004, *p* = 0.002, *p* = 0.017, respectively). No differences were observed in the percentage of motile spermatozoa after treatment between all tested glucose concentrations. No differences were observed in the percentage of motile spermatozoa between those without glucose and the T = 0 h group.

### 3.3. The Main Metabolites Produced during Human Sperm Capacitation Are Lactate, Acetate, and Malate

**Figure 3:**
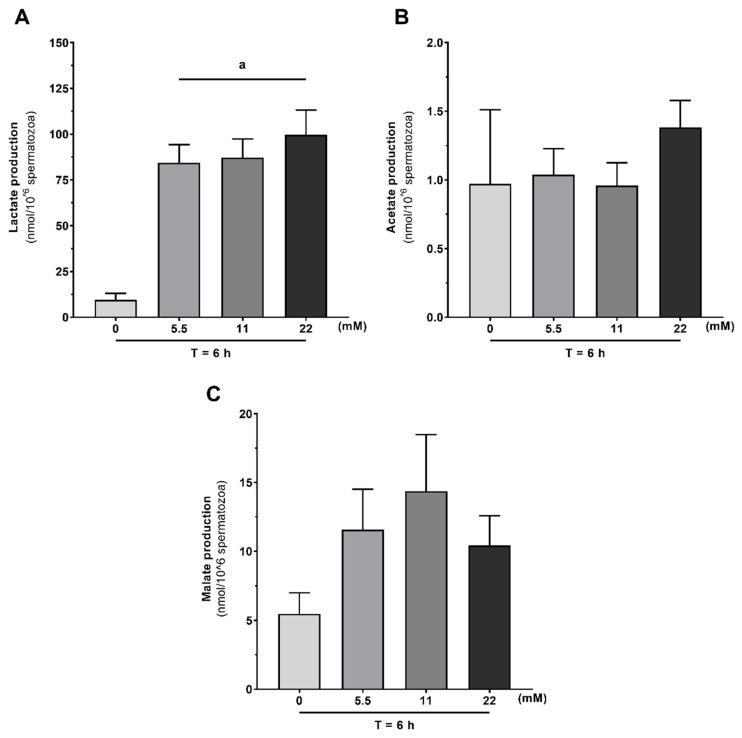
Production of lactate (**A**), acetate (**B**), and malate (**C**) by human spermatozoa 6 h after incubation without or with glucose (0, 5.5, 11, and 22 mM) under capacitation conditions. Results are expressed as mean ± SEM (*n* = 15 for each condition). Significantly different results are expressed as: (**a**) Relative to 0 mM glucose group (T = 6 h).

We analyzed ^1^H-NMR spectra of the media in order to evaluate the metabolic response of human spermatozoa to glucose stimuli and in the absence of exogenous energy substrates under capacitation conditions. Lactate (Figure 3A), acetate (Figure 3B), and malate (Figure 3C) were the main metabolites identified. All glucose concentrations led to spermatozoa lactate production (84.3 ± 9.9 nmol/10^6^ spermatozoa; 87.2 ± 10.2 nmol/10^6^ spermatozoa; 99.8 ± 13.4 nmol/10^6^ spermatozoa, after treatment with 5.5, 11 and 22 mM of glucose, respectively). Interestingly, human spermatozoa produced lactate even in the absence of glucose or other exogenous energetic substrates, though at a lower concentration (10.0 ± 3.0 nmol/10^6^ spermatozoa; *p* < 0.001). No differences were observed concerning acetate production (1.0 ± 0.5 nmol/10^6^ spermatozoa; 1.0 ± 0.2 nmol/10^6^ spermatozoa; 1.0 ± 0.2 nmol/10^6^ spermatozoa; 1.4 ± 0.2 nmol/10^6^ spermatozoa after treatment with 0, 5.5, 11 or 22 mM of glucose, respectively) or malate (5.4 ± 1.5 nmol/10^6^ spermatozoa; 11.6 ± 2.9 nmol/10^6^ spermatozoa; 14.4 ± 4.1 nmol/10^6^ spermatozoa; 10.4 ± 2.2 nmol/10^6^ spermatozoa after treatment with 0, 5.5, 11 or 22 mM of glucose, respectively) among the studied groups.

### 3.4. Mitochondrial Activation during Human Sperm Capacitation Does not Require Glucose Presence

**Figure 4:**
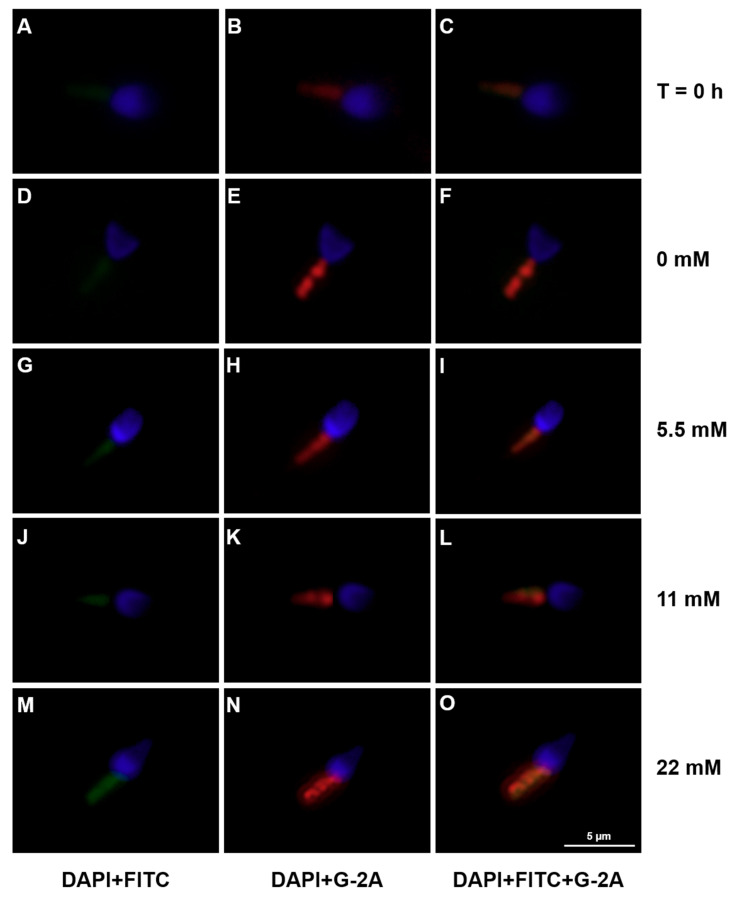
Analysis of JC-1 J-aggregates (red) or monomers (green) in human spermatozoa at baseline (T = 0 h) and without or with glucose (0, 5.5, 11, and 22 mM) stimuli following 6 h in capacitation conditions, by fluorescence microscopy. In (**A**,**D**,**G**,**J**,**M**) are represented JC-1 monomers (green, FITC filter) and nuclei (DAPI filter) for T = 0 h group and glucose concentrations (0, 5.5, 11 and 22 mM), respectively. In (**B**,**E**,**H**,**K**,**N**) are represented JC-1 J-aggregates (red, G-2A filter) and nuclei (DAPI filter) for T = 0 h group and glucose concentrations (0, 5.5, 11 and 22 mM), respectively. In (**C**,**F**,**I**,**L**,**O**) are represented the overlap between JC-1 J-aggregates and monomers for T = 0 h group and glucose concentrations (0, 5.5, 11 and 22 mM), respectively.

**Figure 5:**
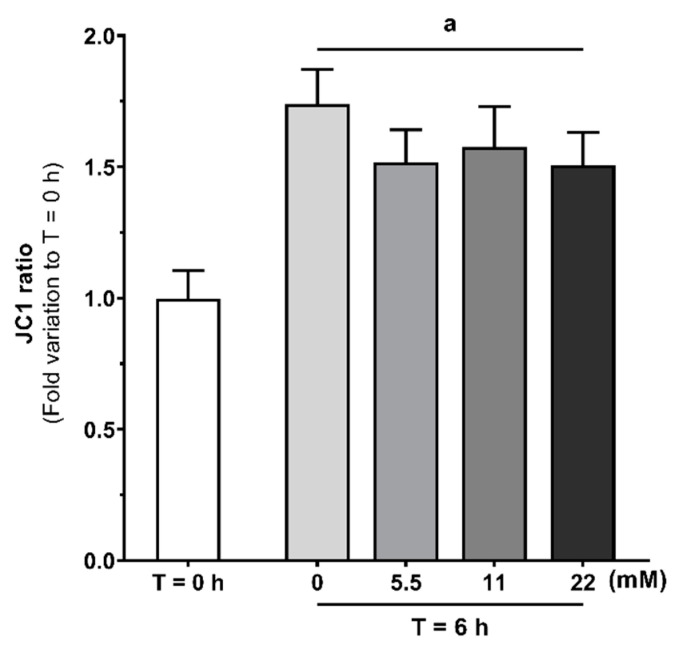
Evaluation of human spermatozoa mitochondrial membrane polarization at baseline (T = 0 h) and without or with glucose (0, 5.5, 11 and 22 mM) stimuli following 6 h in capacitation conditions, using the dye JC-1. Results are expressed as mean ± SEM (*n* = 15 for each condition). Significantly different results are expressed as: (**a**) Relative to T = 0 h group.

The increased motility observed during human sperm capacitation is reported to be supported by increased mitochondrial activity. In order to study the effects of increasing glucose concentrations (5.5, 11 and 22 mM) or absence of glucose or other exogenous energy substrates on human spermatozoa mitochondrial activity during capacitation, we used the JC-1 dye. JC-1 J-aggregates/monomers ratio was used as an indicator of mitochondrial membrane polarization, where an increased/decreased ratio was considered indicative of hyperpolarization/depolarization, respectively. The fluorescence of JC-1 J-aggregates or monomers was firstly observed and captured on a Nikon Eclipse E400 microscope at a 1000× magnification (Figure 4). Herein, we observed that all tested conditions exhibited a more intense fluorescence of J-aggregates (red) in comparison to monomers (green). To confirm this observation, the ratio of JC-1 J-aggregates/monomers was measured using a microplate reader (Figure 5). We observed an increased JC-1 ratio on all tested glucose concentrations (1.52 ± 0.12; 1.57 ± 0.15; 1.51 ± 0.12 after treatment with 5.5, 11 or 22 mM of glucose, respectively) as compared to the T = 0 h group (1.00 ± 0.11; *p* < 0.001), which indicates mitochondrial hyperpolarization. No differences were observed in mitochondrial membrane potential among all the tested glucose groups. We also observed an increased JC-1 ratio in the group treated in the absence of glucose or any other exogenous energetic substrates (1.74 ± 0.13) as compared to the T = 0 h group (*p* < 0.001).

### 3.5. Human Sperm Capacitation Leads to Increased Endogenous ROS Production without Inducing Oxidative Stress-Related Damage

**Figure 6:**
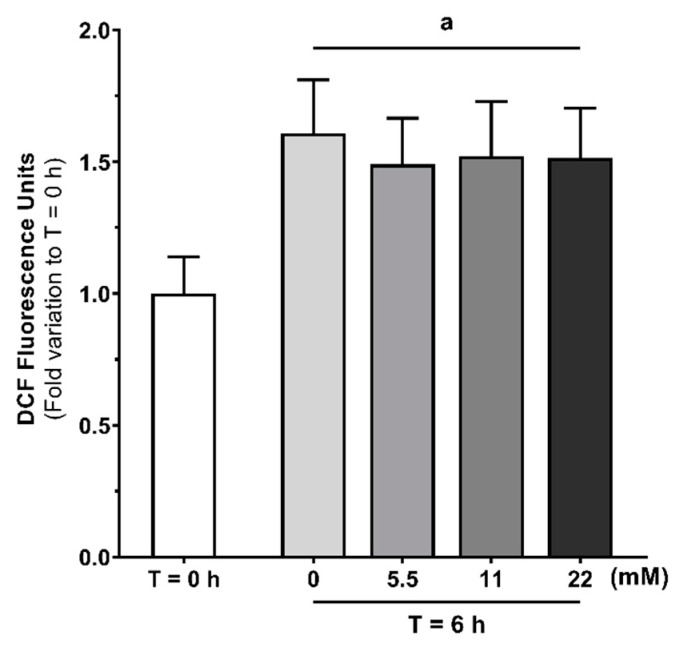
Evaluation of endogenous ROS production in human spermatozoa at baseline (T = 0 h) and without or with glucose (0, 5.5, 11 and 22 mM) stimuli following 6 h in capacitation conditions, using the fluorogenic dye CM-H2DCFDA. Results are expressed as mean ± SEM (*n* = 15 for each condition). Significantly different results are expressed as: (**a**) Relative to T = 0 h group.

Since increased mitochondrial activity was observed, we sought to evaluate the endogenous ROS production using the fluorogenic dye CM-H2DCFDA (Figure 6). Increased endogenous ROS production was observed on all tested glucose concentrations (1.49 ± 0.18; 1.52 ± 0.21; 1.52 ± 0.19 after treatment with 5.5, 11, or 22 mM of glucose, respectively) as compared to the T = 0 h group (1.00 ± 0.14; *p* < 0.001). No differences in spermatozoa endogenous ROS production after treatment with all tested glucose concentrations were observed. Nevertheless, an increase in endogenous ROS production was detected on spermatozoa treated in the absence of glucose or other exogenous energy substrates (1.61 ± 0.20) as compared to the T = 0 h group (*p* < 0.001).

**Figure 7:**
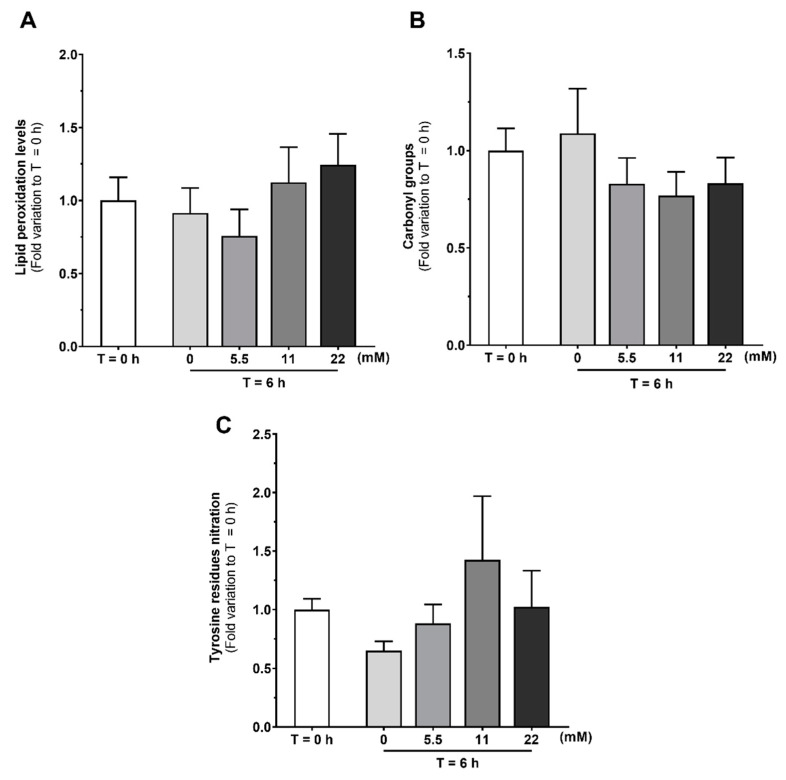
Oxidative stress-induced damage in human spermatozoa at baseline (T = 0 h) and after 6 h incubation without or with glucose (0, 5.5, 11, and 22 mM) under capacitation conditions on lipid peroxidation (**A**), carbonyl group formation (**B**), and tyrosine residues nitration (**C**) in human spermatozoa. Results are expressed as mean ± SEM ((**A**,**B**) *n* = 15 for each condition, (**C**) *n* = 8 for each condition).

Increased ROS production can be associated with oxidative stress-induced damage. Thus, we evaluated the levels of lipid peroxidation (Figure 7A), protein oxidation through determination of protein carbonyl groups content (Figure 7B) and protein nitration (Figure 7C) in human spermatozoa by slot blot technique. Although increased endogenous ROS production was observed, no alterations were detected in spermatozoa lipid peroxidation (0.91 ± 0.17; 50.76 ± 0.18; 1.12 ± 0.24 and 1.25 ± 0.21 after treatment with 0, 5.5, 11 or 22 mM of glucose, respectively), protein oxidation (1.09 ± 0.23; 50.83 ± 0.13; 0.77 ± 0.12; 0.83 ± 0.13 after treatment with 0, 5.5, 11 or 22 mM of glucose respectively), or protein nitration (0.65 ± 0.09; 0.88 ± 0.16; 1.44 ± 0.56; 1.01 ± 0.31 after treatment with 0, 5.5, 11 or 22 mM of glucose, respectively) as compared with the levels detected in spermatozoa of the T = 0 h group (1.00 ± 0.16, 1.00 ± 0.11, 1.00 ± 0.09, respectively).

## 4. Discussion

Human sperm capacitation is a complex step necessary for successful oocyte fertilization. Capacitation is characterized by numerous functional alterations in spermatozoa. Those are mostly characterized by mass phosphorylation of proteins involved in signal transduction and increased oxidation of energetic substrates. In order to achieve this state, it is reported that spermatozoa require glucose as an energetic substrate [8]. Glucose is regarded as the essential hexose for human spermatozoa optimal capacitation and fertilization, supporting their hyperactivated motility [8]. For instance, fructose is present at higher concentrations in the human seminal fluid (mean concentration of 13.88 mM [29]) but does not lead to a complete sperm capacitation [30,31]. Although human seminal fluid contains low glucose amounts (mean concentration of 0.41 mM [32]), in the female reproductive tract sperm cells are exposed to higher glucose concentrations. The human cervical mucus presents a mean 5.2 mM of glucose, which may peak 8.25–11 mM during ovulation [33,34]. Together with a high concentration of HCO_3_^−^, albumin, and Ca^2+^, human sperm capacitation is then promoted [1].

In this work, we studied the effects of increasing glucose concentrations or the absence of glucose or any other energetic substrate, on human sperm capacitation. For this purpose, human spermatozoa were incubated under in vitro capacitation conditions for 6 h without glucose or with increasing glucose concentrations (0, 5.5, 11, and 22 mM) in the absence of any other energy substrate. We observed that exposure to glucose maintained human spermatozoa viability over time after treatment with all studied glucose concentrations, whereas the group without glucose had a reduced percentage of viable spermatozoa. Thus, our results support the literature data highlighting glucose as essential for human spermatozoa viability maintenance over time [35]. In addition, we observed that elevated tyrosine residues phosphorylation levels was only present in groups exposed to glucose, which is a common biomarker of sperm capacitation [36]. These results also support the literature data that suggests glucose to be essential to promote human sperm capacitation [2]. Thus, our data corroborates previous studies findings that glucose is pivotal for human spermatozoa viability and capacitation. Notably, no differences were observed between the several glucose concentrations, which lead us to conclude that glucose stimuli are necessary to trigger human spermatozoa capacitation but this phenomenon is not glucose concentration-dependent.

As a direct consequence of sperm capacitation, there is an increased conversion of energy substrates into mechanical energy, resulting in sperm hyperactivation and increased motility. Hyperactivation is essential for human spermatozoa to swim through the endometrium and oviductal mucus, while oocyte zona pellucida penetration upon acrosome reaction is also necessary [7]. We analysed total motility and observed that the percentage of motile spermatozoa increased in groups exposed to glucose in comparison to the reference group (baseline) or to the no glucose group. These results corroborate the previous results on capacitation, rendering glucose as necessary to initiate the process leading to increased sperm motility. Although we observed an increased motility, we were not able to identify hyperactivated sperm cells with this method. Analysis of hyperactivated human spermatozoa and their motility with a Computer Assisted Sperm Analyzer (CASA) system will be addressed in future research. Interestingly, the absence of energy substrates during capacitation did not decrease spermatozoa motility after 6 h, which is reported to occur when spermatozoa are not incubated under capacitation conditions [37]. Thus, our results may suggest the likely presence of other endogenous energy source able to sustain human spermatozoa motility and whose metabolism could be activated through the ionic environment conferred by the capacitation medium. Notably, our results also suggest that high glucose concentrations, such as two times the peak concentrations found in the female reproductive tract, are not detrimental for human spermatozoa. This is in agreement with studies suggesting that human spermatozoa are highly resistant to glucose toxicity, supporting concentrations up to 50 mM without noticeable adverse effects on sperm parameters [38]. Nonetheless, the mechanisms by which human spermatozoa are able to support such high glucose concentrations without inducing toxic effects remains to be explored.

Both sperm capacitation and hyperactivation require increased energy substrate oxidation for maintenance. Herein, we analysed human spermatozoa metabolism through quantification of metabolites in spermatozoa media by ^1^H-NMR. We were able to identify and quantify the production of lactate, acetate, and malate by spermatozoa. Lactate was the main metabolite produced by human spermatozoa and it was produced in higher amounts after glucose exposure, though there were no differences across glucose concentrations. Lactate was previously reported as the main metabolite produced by human spermatozoa [39]. In addition, human spermatozoa are able to further metabolize lactate into pyruvate and acetate [39,40], which is corroborated by our results that show spermatozoa acetate production after glucose treatment. We were also able to identify and quantify malate in the media. Malate is a citric acid intermediate that also takes part on the malate-aspartate shuttle. Malate is known to increase mitochondrial uptake of carboxylic substrates, leading to an increased mitochondrial activity [41]. Interestingly, the addition of malate to the capacitation media is reported to improve lactate and pyruvate oxidation, while also being required for maximal oxygen uptake [42,43,44]. Although the reason for human spermatozoa to secrete malate during capacitation remains to be elucidated, we hypothesize that its secretion and uptake may be necessary for optimal mitochondrial activity of the surrounding spermatozoa. We also observed that human spermatozoa were able to produce lactate, acetate, and malate even in the absence of glucose or any other energy substrate. It is possible that some energy substrates from the seminal plasma could still be present in the samples even after the washing procedure but that would occur at residual concentration and in all samples. Conversely, these results could also further suggest the hypothesis that human spermatozoa are likely to rely on other endogenous energy sources to sustain motility over time. Taken together, our results support the current hypothesis that human spermatozoa metabolism is highly dynamic and dependent on the energy substrate available [11].

Sperm bioenergetics and the main contributor for ATP production are still under debate. Both glycolysis and mitochondrial respiration are activated and contribute for spermatozoa capacitation and motility [45]. Still, some authors defend glycolysis to be the main ATP source in human spermatozoa [12], while others defend that mitochondrial respiration plays a major contribution for ATP production [13,14,15]. Human sperm mitochondria are dynamic organelles that exhibit some peculiarities. For instance, mitochondria suffer morphological changes during capacitation. Vorup-Jensen et al. reported that under capacitation conditions, mitochondria of human spermatozoa increase their volume and change towards a more loosely wrapped morphology [46]. Additionally, several studies reported that mitochondrial respiration highly increases during capacitation [47,48]. Based on these data, some authors hypothesized that the increase in mitochondrial respiration is associated with the presence of oxidizable substrates in the capacitation medium. In this work, we observed an increase in JC-1 J-aggregates/monomers ratio when human spermatozoa were exposed to glucose in capacitation conditions, which indicates mitochondria membrane hyperpolarization. These results are supported by data in the literature, reporting that in vitro human spermatozoa capacitation led to a peak in O_2_ consumption and increased mitochondrial respiration capacity [16]. In the same study, Stendardi et al. also reported that the high mitochondrial respiratory efficiency remained stable after capacitation, suggesting that mitochondria activity supported hyperactivated motility. However, we also observed an increase JC-1 J-aggregates/monomers ratio after 6 h incubation without glucose or any other energy substrate in the capacitation medium, which still indicates mitochondria membrane hyperpolarization. To the best of our knowledge, this is the first time that human sperm mitochondria activation is described to occur in capacitation medium even in the absence of glucose or any other known exogenous energy substrate. These results suggest that mitochondria activation during human sperm capacitation is dissociated from exogenous glucose stimuli. Thus, we hypothesize that human sperm capacitation could occur as a two-step process. The classic description of sperm capacitation highlights that a high concentration of HCO_3_^−^ stimulates the activity of adenylyl cyclase, which cause an increase in cAMP. Then, cAMP activates protein kinase A (PKA) and triggers spermatozoa capacitation, in the presence of high amounts of ATP produced through glucose oxidation [49]. However, our results provide evidence that spermatozoa exhibit mitochondria activation in a way independent of glucose stimuli. Thus, we hypothesize that mitochondrial activation is the first step to occur during human sperm capacitation, which is mostly supported by the ionic environment established. Glucose would only be necessary for a second step, where high ATP production through glycolysis and mitochondrial respiration occurs and there is a subsequent mass phosphorylation of proteins needed for spermatozoa capacitation and hyperactivated motility. In support to our hypothesis, we also observed increased endogenous ROS production in all tested conditions, even in the absence of glucose. These results provide further evidence for an increased mitochondrial activity independently of glucose or any other energy substrate. Moreover, our results shed some light on possible sperm mitochondria functions other than ATP production through oxidative phosphorylation. Mitochondria are a known source of ROS, being reported to convert 0.2–2% of the oxygen uptake into ROS [18,50]. In small concentrations, ROS are hypothesized to play a physiological role and intervein as regulators in multiple signaling pathways [51,52]. On the other hand, increased ROS production may lead to oxidative stress-induced damage, which is reported to be a common cause for male infertility [53]. For instance, increased ROS production causes DNA fragmentation or lipid peroxidation, which compromises the membrane and impairs mitochondrial activity [53,54]. In fact, antioxidants have been proposed as adjuvants for sperm culture medium, being beneficial for spermatozoa viability and motility maintenance [55,56,57]. Since increased ROS production can lead to oxidative stress-induced damage, we also analyzed lipid peroxidation, protein oxidation, and protein nitration. Interestingly, we were unable to find any evidence of oxidative stress-induced damage, although increased endogenous ROS production was observed. As increased oxidative stress-related damage is reported as the main cause for hyperglycemia-induced damage on pancreatic beta cells [58], the absence of high oxidative stress-induced damage could further explain the high glucose tolerance of human spermatozoa. Taken together, our results suggest that increased ROS production is a product of increased mitochondrial activity and may take part of a finely regulated signaling pathway that modulates or even activates human sperm capacitation. Although the role of endogenous ROS in sperm during capacitation is still a matter of debate, it is reported that spermatozoa incubation under low H_2_O_2_ concentrations improves capacitation, hyperactivation, and acrosome reaction [59]. In addition, there is data evidencing the role of nitric oxide as a signaling molecule for capacitation [60,61]. Still, the role of endogenous ROS as a signaling agents for the fertilization process are still poorly understood and more data are needed to clarify these molecular mechanisms.

In conclusion, our results provide further evidence that glucose is essential for complete capacitation of human spermatozoa, although its stimuli seem to be dissociated from mitochondrial (hyper)activation. We were able to demonstrate that under capacitation conditions, human spermatozoa mitochondria are active and producing ROS even in the absence of glucose or any other energy substrates. Taken together, we propose that human sperm capacitation occurs as a two-step process. On the first step, sperm mitochondria become active and produce ROS as signaling intermediates. As a second step, and when high glucose concentrations are available, such as occurs within the female reproductive tract, mitochondrial respiration increases to sustain the high ATP demands necessary for mass protein phosphorylation and hyperactivated motility. Although our hypothesis constitutes a paradigm shift on the physiology of human sperm capacitation, further studies are needed for its clarification. In addition, the role of the mitochondria and ROS as signaling intermediates during capacitation demands further research.
